# Confidence to Self‐Manage in Diabetes: An All‐Wales Cross‐Sectional Population Study

**DOI:** 10.1155/jdr/5566273

**Published:** 2026-04-03

**Authors:** Jasmine Rollings, Nichola M. Thuvesholmen, Katherine E. Woolley, Sarah Puntoni, Sally Cox, Julia Platts, Alexander Shaw, Christian Newman, Kathleen Withers

**Affiliations:** ^1^ CEDAR, NHS Wales Cardiff and Vale University Health Board, Cardiff, UK; ^2^ NHS Digital Health and Care Wales, Cardiff, UK; ^3^ Value Transformation, NHS Wales Performance and Improvement, Wales, UK; ^4^ Welsh Value in Health Centre, Llantrisant, UK; ^5^ NHS Wales Cardiff and Vale University Health Board, Cardiff, UK; ^6^ Hywel Dda University Health Board, Carmarthenshire, UK; ^7^ Cardiff University, Cardiff, UK, cardiff.ac.uk

## Abstract

**Introduction:**

Diabetes is a leading cause of morbidity and mortality in Wales, and concerningly, the prevalence of diabetes and the associated healthcare costs continue to rise. Effective self‐management is essential in diabetes care to avoid poor outcomes; however, little is known about how confidence to self‐manage impacts outcomes.

**Research and Design:**

This study examines the association between confidence to self‐manage and wellbeing and emergency and elective admissions in a large sample of people with diabetes surveyed in Wales over the age of 45 years (*N* = 2941). Where possible, multilevel regression modelling was used to account for patients being nested within GP practises; otherwise, logistic regression modelling was applied.

**Results:**

After controlling for various individual and situational factors, confidence to self‐manage was found to be strongly associated with better wellbeing (*B* = 12.43; 95% CI: 11.19–13.67) and with reduced odds of having an emergency hospital admission (OR = 0.79; 95% CI: 0.65–0.95) but not an elective hospital admission (OR = 0.95; 95% CI: 0.81–1.13) in the year following survey completion. Other individual level factors such as being unable to work and having more chronic conditions were associated with poorer wellbeing and having an emergency admission.

**Conclusions:**

These findings indicate that higher levels of confidence to self‐manage were associated with better wellbeing and reduced odds of emergency hospital admission among diabetic people aged over 45 years in Wales. Therefore, healthcare providers, policymakers and patients should engage with and develop strategies to enhance confidence and self‐management in diabetes.

## 1. Introduction

Type 1 and Type 2 diabetes are leading causes of morbidity, mortality and high healthcare resource usage (e.g., foot surveillance, hospital admission and prescriptions) in Wales. Over 207,000 people—around one in 13—in Wales have a diabetes diagnosis [1]. Across the United Kingdom, 90% of cases are Type 2, 8% Type 1 and 2% gestational or other types of diabetes. Many more are believed to be undiagnosed [1]. Approximately 10% of the NHS UK budget is spent on diabetes, with the majority spent on treating complications such as ischaemic heart disease, retinopathy, neuropathy and renal failure [2]. People with diabetes are more likely to be admitted to hospital than those without; in 2021/22, 11% of all admissions involved patients with diabetes [3]. Diabetes prevalence is rising, especially for Type 2 diabetes, which has been associated with an increase in unhealthy diets, sedentary lifestyles, an ageing population and widening inequalities [4]. In Wales, prevalence increased by 40% between 2009/10 and 2021/22, representing a challenge for the health service [3].

The diabetes prevention plan communicated in Public Health Wales Long‐Term Strategy 2023–2035 [5] documents primary (wider determinants of health, psychosocial determinants and commercial and behavioural determinants), secondary (early detection and intervention) and tertiary (treatment and care) areas of prevention. A reduction in health events and poor health outcomes resulting from diabetes can be achieved through modifiable risk factors (e.g., education, patient behavioural changes and self‐management). For example, preventing the development of and reversing Type 2 diabetes can be achieved through appropriate lifestyle changes. Equally, adequate glucose control can be maintained through appropriate self‐management to prevent diabetic emergencies and long‐term health complications. Self‐management generally refers to the day‐to‐day management of chronic conditions by individuals and is theoretically constructed of three components: having sufficient knowledge about the condition (e.g., performing healthy behaviours), performing relevant management behaviours (e.g., managing acute episodes and communicating with healthcare professionals) and applying skills for adequate psychosocial coping (e.g., addressing the emotional impact of illness) [6]. To engage in health‐related behaviours [7], individuals require a high level of self‐efficacy, which is defined as a person’s confidence in their capacity to perform specific tasks successfully.

Diabetes is a condition that requires continuous self‐management (e.g., blood glucose self‐monitoring, making informed food and lifestyle choices), as individuals spend most of their time outside direct contact with healthcare providers, and such activity can be complex and demanding. As poor health outcomes can develop quickly because of inadequate self‐management, UK and international policies have emphasised the role of self‐management in chronic disease. Organisations such as the UK Department of Health [8, 9] and the International Diabetes Federation highlight the need for individuals with diabetes to be empowered and supported in making informed decisions about their health, rather than focusing solely on a healthcare provider–driven approach. With the increasing shift towards value‐based healthcare in Wales and wider reliance on the use of patient‐reported outcomes as well as clinically reported outcomes, there needs to be a greater understanding of how patient empowerment can be supported within the healthcare system to enable individuals to have the necessary skills and confidence to self‐manage effectively.

The literature identifies various enablers and barriers to self‐management within the diabetic population [10] and highlights the role of self‐management in improving diabetes‐related health outcomes [11]. Furthermore, there is some evidence to suggest diabetic self‐efficacy, or general self‐efficacy, is linked to diabetic distress and psychological wellbeing in people with diabetes in Saudi Arabia and Australia [12–14]. Confidence to self‐manage is closely related to self‐efficacy but reflects a broader, more applied confidence in managing one’s own health and interactions with health services rather than confidence in performing specific tasks. Generally, there is a paucity of evidence from a UK perspective of the association between self‐management and health and wellbeing outcomes in people with diabetes, nor are the authors aware of any previous publications which outline the individual and situational socio‐demographic characteristics of individuals with diabetes in Wales. This work is important as it may shed light on key social determinants of chronic illness and identify risk or resilience factors that are vital to improving health in Wales. Therefore, in this cross‐sectional national study, drawing on a large, population‐level dataset that links survey responses with administrative hospital data, we investigate the relationship between confidence to self‐manage, patient‐reported wellbeing and hospital admissions (emergency and elective) among individuals with diabetes in Wales.

## 2. Methods

### 2.1. The Population Health Survey

This study is a secondary analysis of the Welsh survey data (the population health survey) collected as part of the Patient‐Reported Indicator Surveys (PaRISs) by the Organisation for Economic Co‐operation and Development (OECD) [15].

PaRIS was designed by an international consortium of researchers in collaboration with stakeholders, participating countries and the OECD. Some items in PaRIS were adapted for each country, for example, education and ethnicity categories. Survey sampling for the OECD PaRIS programme was targeted at GP practises in Wales, with sampling undertaken by CEDAR, an NHS research organisation. Out of a total of 385 practises in Wales, a stratified sample of 199 were selected for inclusion in the Wales population survey. The stratified random sample of GP practises was undertaken to ensure the sample would be as representative of the Welsh population as possible. Patients over 45 years were randomly selected from the 199 GP practises by Digital Health and Care Wales (DHCW) colleagues using the Welsh Demographic Service, a system which includes all patients registered with a GP in Wales. In Wales, participants were aged 46 and over, whereas for other PaRIS countries participants were aged 45 years and over.

Patient survey data was collected July–October 2023, with a response rate of 24% (25,838/109,600). Completion was via paper, online, or phone, with the option to respond in Welsh in accordance with the Welsh Language (Wales) Measure 2011 [16]. Additional language support was available by helpline.

More details on the international OECD PaRIS survey [15] and the PaRIS sampling methodology applied in Wales are available elsewhere [17, 18], and further detail is provided in File S1. The current study is a secondary analysis of the population health survey, as such the authors did not determine the inclusion criteria of the sample, nor the items included in the questionnaire.

### 2.2. Diabetes Cohort

Of 25,839 survey participants, 2,942 (11%) reported that they had either Type 1 or Type 2 diabetes, which formed the diabetic sample in this study. The sample size corresponds closely to the proportion of the population of Wales with a diabetes diagnosis as reported in the Welsh Primary Care Disease Registers [19].

### 2.3. Linkage to Other Data Sources

Data linkage between the Welsh Population Health Survey and nationally held datasets was conducted by DHCW using participants’ NHS numbers. Survey data was linked to reference data within the national data warehouse to assign deprivation deciles to each participant using the Welsh index of multiple deprivation (WIMD) 2019 [20]. Survey records were also linked to the Admitted Patient Care (APC) dataset [21] to identify whether participants had any hospital admissions in the 12 months following survey completion.

Linked datasets were not accessible outside of DHCW; analysis was performed by authors within this organisation.

### 2.4. Wellbeing (Primary Outcome)

The primary outcome in this study was wellbeing, measured using the five‐item World Health Organization Well‐Being Index (WHO‐5) [22]. Participants were asked to indicate for each of the five statements how they felt over the past 2 weeks using a six‐point Likert scale ranging from 0 = “*at no time*” to 5 = “*all of the time*.” The sum of all raw scores ranged from 0 to 25; by multiplying by four, the possible total percentage scores ranged from 0 to 100. The WHO‐5 is widely used and deemed to be psychometrically sound [23, 24].

### 2.5. All‐Cause Emergency and Elective Hospital Admissions (Primary Outcome)

Hospital admission data from the 12 months following each participant′s survey completion date was extracted. Binary variables for whether a participant had an emergency or elective admission (yes/no) for any reason were derived.

### 2.6. Confidence to Self‐Manage (Variable of Interest)

Several questionnaire items concerned confidence to self‐manage; therefore, items were combined into a composite score to represent overall confidence to self‐manage (see Table 1 for details).

**Table 1:** Items that formed the confidence to self‐manage score.

Item	Question	Source	Responses
1	How confident are you that you can manage your own health and wellbeing?	Person‐Centred Coordinated Care Experience Questionnaire (P3CEQ)	Each item had the following response options:(1) Very confident(2) Confident(3) Somewhat confident(4) Not confident at allItem 5 had an additional response option:(1) Not applicable
2	How confident are you that you can follow instructions from health care professionals about how you should care for yourself at home?	Medicare Patient Engagement Questions	
3	How confident are you that you can follow instructions from health care professionals about how to change your habits or lifestyle?	Medicare Patient Engagement Questions	
4	How confident are you that you can identify when it is necessary for you to get medical care?	Medicare Patient Engagement Questions	
5	How confident are you that you can identify when you are having side effects from your medications?	Medicare Patient Engagement Questions	

This novel scale was created by scoring each question from 0 to 3 (0 being the *least confident* and 3 being the *most confident*). Item 5 had an additional response option ’not applicable’ as not all participants would be taking medication; this was set to missing if selected and therefore not included in the overall score. The score was then calculated from the mean of all five items. Therefore, the possible range of scores was 0–3. The confidence to self‐manage scale demonstrates good internal consistency in this sample (Cronbach’s *α* = 0.84 [*C* *I* = 0.825–0.846]), meaning the items are reliably measuring the same construct. The narrow confidence interval suggests robustness in the estimate. See File S4 for item correlation matrix. A maximum likelihood exploratory factor analysis specifying a one‐factor solution was undertaken, consistent with the theoretical structure of the scale and the observed item correlations. The analysis showed that all five items loaded meaningfully onto a single factor, with loadings ranging from moderate to strong and the factor explaining just over half of the total variance (see File S5). Face validity of the items was established through conversation with clinical professionals.

### 2.7. Individual and Situational Level Confounding Factors

Individual level confounding factors included self‐reported gender (male, female and other), age (45–49, 50–54, 55–59, 60–64, 65–69, 70–74, 75–79, 80–84 years old and 85 years or older), highest level of education (no formal qualifications, GCSE/NVQ/equivalent and A level/degree/higher or equivalent), ethnicity (Welsh, English, Scottish Northern Irish or British and other) and number of chronic conditions (none, one, two, three or more). Due to the lack of variability in ethnicity data (90% Welsh, English, Scottish Northern Irish or British and 4% nonresponse), this was not included in the statistical models. Due to small numbers in the ‘other’ gender category, these participants were not included in the statistical models. See Files S2 and S3 for additional information on survey categories.

Situational level confounding factors included household net income (up to £22,425, £22,425–£37,375 and £37,375+), occupation (employed retired unable to work and other), area of residence (town or suburb, rural area and city) and WIMD (80%–100% [most deprived], 60%–80%, 40%–60%, 20%–40% and 0%–20% [least deprived]). WIMD is a measure of relative deprivation for lower layer super output areas (LSOAs) in Wales by ranking LSOAs in Wales from most to least deprived, this is reflected in deciles (i.e., 0%–20% ranking represents being in the least deprived LSOAs). Area of residence was not included in the analyses due to being highly related to WIMD and health board.

### 2.8. Data Analysis

Data cleaning and analysis was performed in R Studio (R studio Version 2024.12.1‐563: R Version 4.4.3). Descriptive data are summarised as *m* *e* *a* *n* ± *s* *t* *a* *n* *d* *a* *r* *d* *d* *e* *v* *i* *a* *t* *i* *o* *n* (for continuous variables) or percentages (for categorical variables). In the descriptive analyses, the diabetic cohort is compared to the population health survey sample of 25,838 participants.

Generalised linear regression modelling was used to assess the association between confidence to self‐manage and wellbeing. Logistic regression modelling was used to study the impact of confidence to self‐manage on hospital admission outcomes. Model comparisons based on model fit statistics were performed to determine the required multilevel structure. Visual inspection of residual plots indicated acceptable distribution in the final models. Variable inflation factors were all below 2.5, suggesting there were no collinearity concerns.

#### 2.8.1. Data Missingness

Initial inspection of data indicated that some potential covariates were missing a large proportion of data, or the variability of responses was low. BMI data, derived from height and weight participant responses, was missing for more than 1/3 of participants, and an even larger proportion were missing income data. BMI and income data are likely to be missing not at random (MNAR); therefore, multiple imputation was not applied. Due to missingness, and because income was related to employment status, BMI and income were not included in the statistical analysis, and a complete case analysis was performed.

#### 2.8.2. Clustering of Data

**Figure 1:**
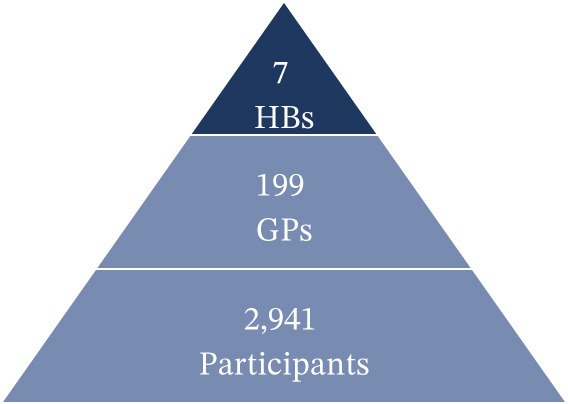
Hierarchical representation of the sample data structure.

Participants belonged to one of 199 GP practises within seven health boards. Therefore, the data was hierarchically clustered—individuals were nested within a GP practise, and GP practise was nested within a Health Board (Figure 1). Either or both grouping factors could have influenced the outcome or the predictors in the analysis, so multilevel modelling (MLM) was used to assess the necessity to account for clustering in the analyses. Due to GP practise being included in the analytical model, area was not included in the analysis to avoid multicollinearity.

### 2.9. Ethics Statement

PaRIS survey data were collected for the purposes of service evaluation and improvement, which, under NHS guidance, does not require formal ethical approval. This secondary analysis was conducted using de‐identified data that does not contain any personal identifiers or sensitive information and therefore did not require additional ethical approval.

## 3. Results

### 3.1. Sample Characteristics

The diabetes sample (a subset of the population health survey sample) had a higher proportion of participants who were male, of older age, had a higher average BMI and were retired or unable to work compared to the population sample (Table 2). In the diabetes sample, the proportion of participants with no formal qualifications was higher and the proportion earning in the highest wage bracket was lower than the population sample. There were no notable differences between samples for area of residence or ethnicity. The diabetes sample has on average lower scores in confidence to self‐manage and WHO‐5 Wellbeing than the total population sample. A larger proportion of the diabetes sample had an emergency (11%) or any hospital admission (22%) and had a higher mean number of hospital admissions in the year after being surveyed. Please see File S6 for the characteristics of the nondiabetic participant population.

**Table 2:** Sample characteristics of the diabetes and population health survey samples.

		Diabetes sample *N* = 2,942		Population health sample *N* = 25,839	
*Outcomes*					
WHO‐5: Wellbeing score		*n* = 2,930		= 25,148	
	Mean (SD)	50.67	24.76	58.46	23.41
Emergency admission (12 months post survey)	*Yes*	345	12%	1,802	7%
Any hospital admission (12 months post survey)	*Yes*	688	23%	4,392	17%
Number of admissions (12 months post survey)	Mean (SD)	0.55	1.91	0.38	1.57
*Variable of interest*					
Confidence to self‐manage score		*n* = 2,940		*n* = 24,893	
	Mean (SD)	1.77	0.65	1.96	0.62
*Individual level characteristics*					
Gender		^∗^		*n* = 23,975	
	Female	1,137	40%	12,672	53%
	Male	1,708	60%	11,286	47%
	Other	^∗^		17	<1%
Age category (years)		*n* = 2,938		*n* = 25,801	
	46–54	316	11%	4,506	17%
	55–64	743	25%	7,591	29%
	65–74	1,009	34%	8,030	31%
	75–84	722	25%	4,693	18%
	85+	148	5%	981	4%
Number of chronic conditions				*n* = 24,662	
	0	—	—	5,753	23%
	1	367	12%	7,733	31%
	2	778	26%	5,758	23%
	3 or more	1,797	61%	5,418	22%
Employment		*n* = 2,834		*n* = 23,918	
	Employed	678	24%	8,821	37%
	Retired	1,783	63%	12,962	54%
	Unable to work	274	10%	1,190	5%
	Other	99	3%	945	4%
Highest education		*n* = 2,631		*n* = 22,646	
	No formal qualifications	731	28%	4,057	18%
	GCSE/NVQ/equivalent	721	27%	5,950	26%
	A level/degree/higher or equivalent	1,179	45%	12,639	56%
Ethnicity		*n* = 2,814		*n* = 23,736	
	Welsh, English, Scottish, Northern Irish or British	2,655	94%	22,568	95%
	Other	159	6%	1,168	5%
BMI		*n* = 2,132		*n* = 18,575	
	Mean (SD)	30.42	6.9	27.70	5.8
*Situation level characteristics*					
Area		*n* = 2,806		*n* = 23,784	
	City	405	14%	3,174	13%
	Town or suburb	1,303	46%	10,872	46%
	Rural	1,098	39%	9,738	41%
Household income		*n* = 1,793		*n* = 16,941	
	Up to £22,425	767	43%	5,821	34%
	£22,425–£37,375	599	33%	5,357	32%
	£37,375 +	427	24%	5,763	34%
WIMD		*n* = 2,942		*n* = 25,827	
	80%–100% most deprived	472	16%	3,135	12%
	60%–80%	700	24%	6,982	27%
	40%–60%	648	22%	5,524	21%
	20%–40%	576	20%	4,191	16%
	0%–20% least deprived	546	19%	5,995	23%

*Note:* Counts and percentage (of those for whom data is available) presented unless stated otherwise. Denominators vary because the variables have different completion rates or come from different sources.

^∗^Suppressed due to small cell counts.

### 3.2. Investigating Health Board and GP Level Effects

MLM is a statistical technique which accounts for clustering in data and can be used to explore the impact of context (e.g., GP practise) on outcomes.

To assess the extent of clustering in the wellbeing model we compared the fit of three unadjusted multilevel regression models: clustering at GP practise level only, Health Board level only and GP clustered within Health Board to a standard regression model. Model fit statistics indicated that including GP practise level improved model fit (intraclass correlation coefficient (ICC) = 0.02, *Δ* *A*IC = 6), but that including Health Board did not (ICC = 0.004, *Δ* *A*IC = 4). As a result, the final wellbeing model in this study was a multilevel linear regression model with nesting at GP level only. For the hospital admission models, many GP practises and health boards had no participants with the outcome of interest (emergency or elective admissions). Consequently, these models were specified as simple logistic regressions without random effects. See File S7 for the model comparisons.

### 3.3. Investigating the Association Between Confidence to Self‐Manage and Wellbeing

The multilevel model accounted for 34% of the variance in wellbeing between individuals in this sample. The ICC was 0.01, suggesting that only 1% of the variance in wellbeing is attributable to differences between GP practises, once other individual factors are included in the model.

**Figure 2:**
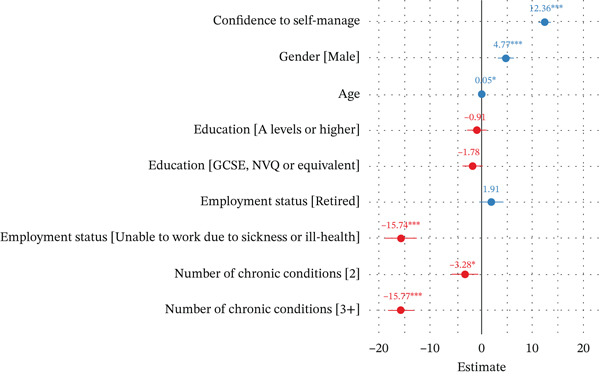
Multilevel linear regression coefficients with confidence intervals (95%) for key predictors of wellbeing (*N* = 2600). *Note:* Blue regression coefficients have a positive association and red have a negative association with the outcome.  ^∗^ *p* < 0.01,  ^∗∗^ *p* < 0.05 and  ^∗∗∗^ *p* < 0.001. Reference categories for categorical predictors: female, no formal qualifications, employed and having one chronic condition. Full model results are available in File S8.

Confidence to self‐manage was an important statistical predictor of wellbeing in people with diabetes, even after accounting for a range of sociodemographic factors, with a one‐unit increase in confidence to self‐manage being associated with a 12.43‐point (95% CI: 11.13–13.60) greater wellbeing score (Figure 2).

Having a higher number of co‐morbidities was associated with lower wellbeing (*B* = −15.77; 95% CI: −18.86 to −13.26). Patients who were employed or retired reported higher wellbeing scores than other employment statuses; compared to being employed, participants who were unable to work had lower wellbeing (*B* = −15.74; 95% CI: −18.86 to −12.62). People who self‐reported as male (*B* = 4.77; 95% CI: 3.17–6.37) and older participants had higher wellbeing (*B* = 0.05; 95% CI: 0.01–0.09). There is little evidence that education had a strong association with wellbeing. Across WIMD groups, wellbeing was lower for the most deprived participants compared to the least deprived participants (*B* = 3.31; 95% CI: 0.51–6.11) and the next most deprived participants (*B* = 3.68; 95% CI: 1.03–6.32).

### 3.4. Investigating the Association Between Confidence to Self‐Manage and All‐Cause Hospital Admissions (Emergency and Elective)

#### 3.4.1. Emergency Admissions (12‐Month Period Post Survey)

**Figure 3:**
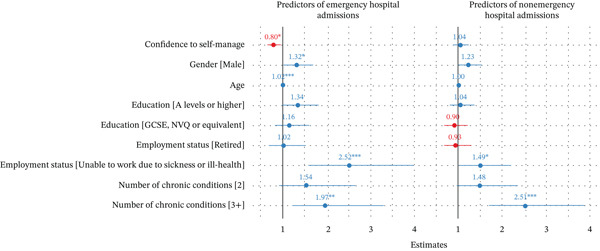
Logistic regression odds ratios with confidence intervals (95%) for key predictors of emergency and elective hospital admissions (*N* = 2600). *Note:* Blue regression coefficients have a positive association and red have a negative association with the outcome.  ^∗^ *p* < 0.01,  ^∗∗^ *p* < 0.05 and  ^∗∗∗^ *p* < 0.001. Reference categories for categorical predictors: female, no formal qualifications, employed, having one chronic condition. Full model results are available in Files S9 and S10.

The adjusted logistic regression results indicated that higher confidence to self‐manage was associated with a 20% decrease in the odds of having an emergency admission (OR = 0.80; 95% CI: 0.66–0.96). Participants who self‐reported as male (OR = 1.32; 95% CI: 1.03–1.70), were in an older age category (OR = 1.02; 95% CI: 1.01–1.02), were unable to work due to sickness (OR = 2.52; 95% CI: 1.60–3.98) and those with three or more chronic conditions (OR = 1.97; 95% CI: 1.24–3.29) were more likely to have an emergency admission (Figure 3).

#### 3.4.2. Elective Admissions (12‐Month Period Post Survey)

There is no evidence that confidence to self‐manage is associated with a significant change in odds of having an elective admission (OR = 1.04; 95% CI: 0.89–1.23). Participants with three or more chronic conditions (OR = 2.51; 95% CI: 1.69–3.86) and those unable to work (OR = 1.49; 95% CI: 1.01–2.19) had increased odds of having an elective admission (Figure 3).

## 4. Discussion

We report that greater confidence to self‐manage is associated with better wellbeing and reduced odds of having an emergency admission in a large sample of people aged over 45 years with diabetes in Wales. These associations remain even after adjusting for several important individual and sociodemographic factors. Wellbeing, while vital in and of itself, is also a fundamental indicator of happiness, mental health, and is associated with a range of other health outcomes and may serve a protective role in health maintenance [25]. Since wellbeing is so closely linked with general health and longevity, and because the international PaRIS findings indicated that Wales has lower self‐reported wellbeing scores compared to other countries included in the survey [15], it is important for policymakers and healthcare providers in Wales to consider how wellbeing outcomes can be improved.

In Wales, recent data reveals a 2% increase in emergency hospital admissions in the year leading to 2022/3 [26]. Reducing emergency hospital admissions is crucial for both individual health outcomes and for optimising healthcare resource use. This is especially important for the diabetic population, as hospitalisation rates are notably higher for individuals with diabetes compared to those without [27]. The current study found that individuals with higher confidence to self‐manage were less likely to experience an emergency admission (for any reason) within the 12 months following the survey. However, this association did not extend to elective admissions (for any reason), possibly because they are less likely to be affected by suboptimal self‐management. Elective admissions are typically scheduled and coordinated by healthcare providers for routine monitoring or planned procedures, meaning that they are less directly influenced by patients’ day‐to‐day self‐management behaviours. In contrast, emergency hospitalisations are more often related to disease exacerbation which are closely linked to variability in daily self‐management—for example, glycaemic control is closely linked to experiencing complications and hospitalisation [28, 29]. This distinction underscores why emergency admissions may be more sensitive to patient confidence to self‐manage than elective admissions.

Confidence to self‐manage is an important part of patient activation, disease management and, pivotally, can be modified and improved. One avenue for improvement is by education and patient empowerment. Evidence shows that self‐management training in diabetes can lead to improvements in outcomes, such as HbA1c reduction [30] and may improve wellbeing in diabetes patients [31]. There has been an emphasis on diabetes patient education programmes in Wales; however, uptake on these courses is low. In 2022, approximately 58% of newly diagnosed Type 2 diabetics in Wales were offered to attend structured education programmes, but less than 2% of the newly diagnosed attended [32]. Findings from the current study can be used to help promote the advantages of self‐management and encourage individuals to attend education courses or reflect on individual management practises. Healthcare providers may wish to concentrate these efforts by using tools such as the Consumer Health Activation Index (CHAI) [33] or the Patient Activation Measure (PAM‐13) [34] to identify individuals with low activation, who may require additional support to self‐manage.

As well as confidence to self‐manage, other individual factors were associated with health and wellbeing outcomes for people with diabetes, after adjusting for other covariates. Having more chronic conditions, even after controlling for other factors, had a negative relationship with wellbeing and participants with more than one condition were more likely to have either type of hospital admission, compared to having a single chronic condition (in this case diabetes). The most prevalent comorbidities in this sample were high blood‐pressure, arthritis and cardiovascular or heart conditions. In fact, only around 12% of the diabetes sample had diabetes as their sole diagnosis. While this study is focused on diabetes, these participants are likely to be managing a range of symptoms and treatments, that may or may not be related to their diabetes diagnosis. Based on this study, patients with low confidence and multiple chronic conditions are key targets for intervention; personalised education (e.g., training on complication management skills) could be used to improve confidence. In the same domain, being unable to work due to sickness or ill‐health had a strong negative association with wellbeing and was associated with greater likelihood of having an emergency admission. Males had better wellbeing than females, a finding consistent with other studies of both people with diabetes [35] and in general populations [36, 37]. And, males were more likely to have either type of hospital admission, which aligns with existing literature on both individuals with and without diabetes [38]. It is important to note that in our adjusted models, these associations represent the relationships between predictors and outcomes after accounting for other factors included in the model, rather than the effect of a predictor alone. Future studies could examine the combined effects of multiple risk factors or explore how these factors interact with confidence to self‐manage.

Overall, there appear to be some sociodemographic differences between the diabetes and the population sample. The diabetic sample was more likely to be male, older, retired or unable to work and have higher BMI. They are also more likely to have no formal qualifications and be in a lower income threshold. For some of these factors there was little evidence of an association with wellbeing or hospital admission outcomes; however, it is possible that factors such as education contribute to confidence to self‐manage. Future work could explore contributory factors in forming confidence and competence in diabetes self‐management.

In this study, we created a novel scale comprising five questions from the OECD PaRIS survey to capture an individual’s confidence to self‐manage. These questions were drawn from two established sources that are currently used to assess patient reported outcomes and experiences: the Person‐Centred Coordinated Care Experience Questionnaire (P3CEQ) [39] and the Medicare Patient engagement questionnaire. The OCED elected to use only the single item from the P3CEQ to reflect confidence to self‐manage in their report [15]. However, this single item was not validated as a standalone measure of confidence to self‐manage. Moreover, single‐item scales are also more susceptible to self‐report errors or random measurement error [40], using a composite scale, such as the one used in this study, can capture a broader perspective of the underlying construct and reduce the impact of reporting errors. Future research would benefit from assessing the extent to which both the single‐item and composite scales accurately capture confidence to self‐manage when compared with validated measures and, ideally, with observable behaviours that indicate confidence to self‐manage.

### 4.1. Limitations

Participants self‐reported having been told by a doctor that they had diabetes, so there is a possibility of reporting error. However, there is a high confidence in this self‐report as the percentage of participants in this sample reporting diabetes, and that in the disease register for Wales with diabetes is very similar. In PaRIS, participants were asked to report if they had diabetes (Type 1 or 2); therefore, distinguishing between the two types is not possible. Future studies should conduct stratified analyses to clarify differences in the role of confidence to self‐manage between diabetes types.

In this study, hospital admissions—whether emergency or elective—are all‐cause admissions. This means that the admissions may or may not have been related to the patient’s diabetes diagnosis. The use of all‐cause admissions is common in diabetes outcomes research [41], as overall health of these patients is clinically relevant and because admissions not coded as diabetes‐related may still be linked to the condition. This may occur due to limitations in clinical coding, which can fail to capture the underlying cause of an admission, or because events are indirectly related to diabetes in ways not reflected in coding data. However, the inclusion of all‐cause admissions may also dilute associations specific to diabetes‐related complications, and findings should therefore be interpreted as reflecting broader health outcomes rather than diabetes‐specific hospitalisation risk. One advantage of the admission data used in this study is the temporal ordering: confidence to self‐manage was measured prior to the outcome, which was up to a year after survey completion. While this still represents an association rather than proof of causality, it allows for a stronger inference in relation to admissions since confidence measurement preceded the outcome. However, this consideration does not apply to wellbeing, which was measured concurrently with confidence. As a result, the wellbeing analysis addresses only whether an association exists between confidence to self‐manage and wellbeing; it does not permit causal interpretation. Longitudinal studies are required to determine whether confidence to self‐manage predicts outcomes, rather than the reverse (e.g., better health status leading to greater confidence).

Confidence to self‐manage is not self‐management behaviour; this paper does not answer whether confidence to self‐manage results in better or worse diabetes self‐management. However, it is likely that confidence to self‐manage is related to self‐management behaviours, as seen in studies linking perceived health competence with health behaviours in older adults, and health‐related quality of life in patients with cardiovascular disease [42, 43]. The PaRIS survey, designed for the general population aged 45 and over, was not specifically focused on individuals with diabetes or diabetes management. As a result, condition‐specific questions were not included, and we cannot conclude that those with greater confidence actually engage in more diabetes‐related self‐management behaviours.

This survey was completed by individuals registered with a selected sample of GP practises. These practises were selected via stratified sampling to be representative of the population in Wales; however, patient participant selection was not curated in the same manner. Consequently, the sample is not guaranteed to be fully representative, although it reflects variation in social deprivation, age distribution and geographical spread. The response rate was approximately 24%, raising the possibility of nonresponder bias, as respondents may differ systematically from nonrespondents in characteristics such as health status or engagement with care. Comparison with census data suggests that the rurality of GP practises and the sex distribution are broadly comparable to the wider population, although there are slight differences in the age distribution [17]. In Wales, PaRIS sampling included participants over the age of 45 years rather than 45 years and over, resulting in a slightly older sample than in other PaRIS countries, potentially reducing comparability. Census data also indicates that the population health survey sample may slightly underrepresent individuals aged 45–65 years [17], which may affect the generalisability of the findings to the wider Welsh population. This is particularly relevant given evidence that subjective wellbeing in English‐speaking countries typically follows a U‐shaped trajectory in later adulthood, with the lowest levels of wellbeing reported among adults aged 45–54 years [25]. It is possible that confidence to self‐manage also differs in this age group; furthermore, the use of broad age categories rather than specific ages limits granularity, which could be addressed in future studies.

## 5. Conclusions

Higher confidence to self‐manage was associated with better wellbeing and reduced odds of having a subsequent emergency hospital admission in a large sample of people aged over 45 years with diabetes in Wales, even when accounting for factors such as age, gender and relative deprivation. Given that effective diabetes care relies on self‐management, individuals with low confidence may be at greater risk of poorer outcomes. Healthcare providers in Wales should prioritise strategies that support self‐management and foster confidence among patients.

## Author Contributions

Writing—original draft: J.R.; methodology: J.R., N.M.T. and K.E.W.; formal analysis and visualisation: J.R. and N.M.T.; data curation: N.M.T., S.C. and A.S.; supervision: S.P., S.C. and K.E.W; conceptualisation and writing—review and editing: all authors.

## Funding

This work was supported by the Welsh Value in Health Centre.

## Disclosure

J.R. presented this manuscript as a poster at the 9th National Patient‐Reported Outcome Measures (PROMs) Research Conference, hosted by the Centre for Patient Reported Outcomes Research (CPROR) at the University of Birmingham, United Kingdom, on 19 June 2025 [44].

## Conflicts of Interest

The authors declare no conflicts of interest.

## Supporting information


**Supporting Information** Additional supporting information can be found online in the Supporting Information section. File S1: Additional information on the OECD PaRIS international survey. File S2: Full list of the education response options that were available to participants in the survey. File S3: Full list of the occupation response options that were available to participants in the survey. File S4: Correlation matrix of confidence to self‐manage scale items. File S5: Factor analysis of confidence to self‐manage scale. File S6: Sample characteristics table for participants with diabetes, participants without diabetes and the overall population health survey sample. File S7. Multilevel model comparisons for wellbeing outcome models. File S8. Multilevel linear regression model results for the Wellbeing model. File S9. Logistic regression model results for the emergency admissions model. File S10: Logistic regression model results for the elective admissions model.

## Data Availability

The data are not publicly available due to privacy or ethical restrictions.
