# Supplementary material for: Confidence to Self‐Manage in Diabetes: An All‐Wales Cross‐Sectional Population Study
Source: J Diabetes Res. 2026 Apr 3;2026:5566273. doi: 10.1155/jdr/5566273 (PMC13051847; doi:10.1155/jdr/5566273)
Supplement: Supplementary file 1 — Supporting Information Additional supporting information can be found online in the Supporting Information section. File S1: Additional information on the OECD PaRIS international survey. File S2: Full list of the education response options that were available to participants in the survey. File S3: Full list of the occupation response options that were available to participants in the survey. File S4: Correlation matrix of confidence to self‐manage scale items. File S5: Factor analysis of confidence to self‐manage scale. File S6: Sample characteristics table for participants with diabetes, participants without diabetes and the overall population health survey sample. File S7. Multilevel model comparisons for wellbeing outcome models. File S8. Multilevel linear regression model results for the Wellbeing model. File S9. Logistic regression model results for the emergency admissions model. File S10: Logistic regression model results for the elective admissions model. [file JDR-2026-5566273-s001.docx]

Supplementary Materials for “Confidence to self-manage in Diabetes: An All-Wales cross-sectional population study”

Appendix 1 – additional information on the OECD PaRIS

The Organisation for Economic Co-operation and Development (OECD) PaRIS is an international programme collecting survey data on outcomes and experiences of people aged 45 years and over, living with chronic conditions across 19 countries including Wales. Within Wales, an OECD PaRIS steering group was established by the Welsh Value in Health Centre (WViHC) to oversee all elements of the project including information governance, communications and engagement and data analysis approaches. The WViHC is accountable to Welsh Government and is part of NHS Wales.

Appendix 2 - Education response categories

- Bachelor's degree/equivalent (NVQ6)
- Master's degree/equivalent (NVQ7)
- PhD /equivalent (NVQ8)
- No formal qualifications (left school before age 11)
- No formal qualifications (left school between age 11 and 14)
- No formal qualifications (left school after age 14)
- GCSEs grades 1-3 or G-D or equivalent (NVQ1)
- Other NVQ2 (e.g., ESOL, music grades 4-5, intermediate apprenticeship)
- GCSEs/ O-levels grades 4 or C and higher (NVQ2)
- A-level, Scottish Higher, International Baccalaureate or equivalent (NVQ3)
- Other NVQ3 (e.g., ESOL, music grades 6, 7 & 8, advanced apprenticeship)
- Access to higher education diploma (level 3)
- Certificate of higher education (CertHE)
- HNC /equivalent (NVQ4)
- Diploma of higher education, foundation degree, HND or equivalent (NVQ5)

Appendix 3. Occupation response options

- Not answered
- Multiple responses
- Question not asked
- Self employed
- In paid employment
- Looking for work
- Looking after the home
- Unable to work due to sickness or ill-health
- Retired
- Student
- Not working and not looking for work
- Apprentice
- Other


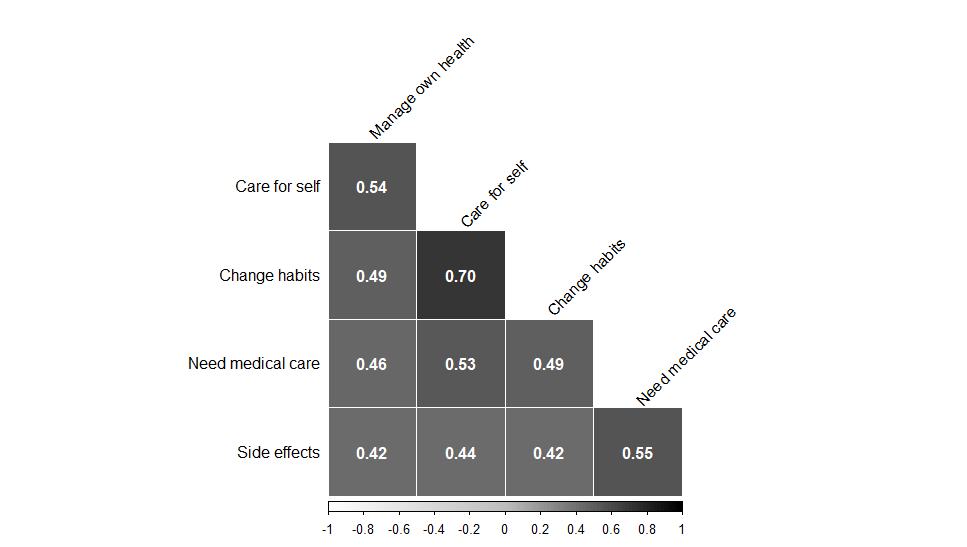
Appendix 4: Correlation matrix of confidence to self-manage scale items

Appendix 5: Factor Analysis

| Item | Loading | Communality (h2) |
| --- | --- | --- |
| Care for self | 0.84 | 0.70 |
| Change habits | 0.79 | 0.62 |
| Need medical care | 0.68 | 0.46 |
| Manage own health | 0.66 | 0.44 |
| Side effects | 0.60 | 0.36 |
|  | | |
| Eigenvalue | | 2.58 |
| Proportion of variance explained | | 0.52 |
| χ² | | 342.82 |
| RMSEA | | 0.15 [90% CI: 0.14–0.17] |
| RMSR | | 0.06 |

Prior to conducting factor analysis, the dataset was inspected for suitability using the Kaiser–Meyer–Olkin measure of sampling adequacy and Bartlett’s test of sphericity. Both indicators supported proceeding with factor analysis.

A maximum likelihood exploratory factor analysis with a one-factor solution was then performed. All five items loaded meaningfully onto the factor, with standardised loadings ranging from 0.60 to 0.84, and the factor accounted for 52% of the total variance (eigenvalue = 2.58). Model fit indices showed mixed evidence for fit: the chi-square test was significant, χ²(5) = 342.82, p < .001, and the RMSEA indicated poor fit (RMSEA = 0.154, 90% CI: 0.14–0.17). However, the RMSR was acceptable (0.06), and the Tucker–Lewis Index indicated adequate but suboptimal fit (TLI = 0.88). Taken together, these results support a single underlying factor.

Appendix 6: Sample characteristics of the diabetic, non-diabetic and overall Population Health samples

|  |  | Diabetes Sample  N = 2,942 | | Non-diabetic Sample  N = 22,897 | | Population Health Sample  N = 25,839 | |
| --- | --- | --- | --- | --- | --- | --- | --- |
| Outcomes | | | | | | | |
| WHO-5: Wellbeing score |  | **n = 2,930** | | **N = 22,218** | | **n = 25,148** | |
|  | *Mean (SD)* | 50.66 | 24.76 | 59.46 | 23.41 | 58.46 | 23.41 |
| Emergency admission  (12 months post survey) | *Yes* | 345 | 12% | 1,456 | 6% | 1,802 | 7% |
| Any hospital admission (12 months post survey) | *Yes* | 688 | 23% | 3,704 | 16% | 4,392 | 17% |
| Number of admissions (12 months post survey) | *Mean (SD)* | 0.55 | 1.91 | 0.35 | 1.52 | 0.38 | 1.57 |
| Variable of interest | | | | | | | |
| Confidence to self-manage score |  | **n = 2,940** | | **N = 22,897** | | **n = 24,893** | |
|  | *Mean (SD)* | 1.77 | 0.65 | 1.99 | 0.61 | 1.96 | 0.62 |
| Individual level characteristics | | | | | | | |
| Gender |  | ***** | | ***** | | **n = 23,975** | |
|  | *Female* | 1,137 | 40% | 11,535 | 55% | 12,672 | 53% |
|  | *Male* | 1,708 | 60% | 9,578 | 45% | 11,286 | 47% |
|  | *Other* | * |  | * |  | 17 | <1% |
| Age category (years) |  |  |  | **n = 22,863** | | **n = 25,801** | |
|  | *46-54* | 316 | 11% | 4,190 | 18% | 4,506 | 17% |
|  | *55-64* | 743 | 25% | 6,848 | 30% | 7,591 | 29% |
|  | *65-74* | 1,009 | 34% | 7,021 | 31% | 8,030 | 31% |
|  | *75-84* | 722 | 25% | 3,971 | 17% | 4,693 | 18% |
|  | *85+* | 148 | 5% | 833 | 4% | 981 | 4% |
| Number of chronic conditions |  |  |  | **N = 21,704** | | **n = 24,662** | |
|  | *0* | - | - | 5,737 | 27% | 5,753 | 23% |
|  | *1* | 367 | 12% | 7,366 | 35% | 7,733 | 31% |
|  | *2* | 778 | 26% | 4,980 | 24% | 5,758 | 23% |
|  | *3 or more* | 1,797 | 61% | 3,621 | 17% | 5,418 | 22% |
| Employment |  | **n = 2,834** | | **N = 21,130** | | **n = 23,918** | |
|  | *Employed* | 678 | 24% | 8,143 | 39% | 8,821 | 37% |
|  | *Retired* | 1,783 | 63% | 11,179 | 53% | 12,961 | 54% |
|  | *Unable to work* | 274 | 10% | 916 | 4% | 1,190 | 5% |
|  | *Other* | 99 | 4% | 892 | 4% | 945 | 4% |
| Highest Education |  | **n = 2,631** | | **N = 20,015** | | **n = 22,646** | |
|  | *No formal qualifications* | 731 | 28% | 3,326 | 16% | 4,057 | 18% |
|  | *GCSE/NVQ/equivalent* | 721 | 27% | 5,229 | 26% | 5,950 | 26% |
|  | *A level/degree/higher or equivalent* | 1,179 | 45% | 11,460 | 57% | 12,639 | 56% |
| Ethnicity |  | **n = 2,814** | | **N = 20,922** | | **n = 23,736** | |
|  | *Welsh, English, Scottish, Northern Irish or British* | 2,655 | 94% | 19,913 | 95% | 22,568 | 95% |
|  | *Other* | 159 | 6% | 1,009 | 5% | 1,168 | 5% |
| BMI |  | **n = 2,132** | | **N = 17,913** | | **n = 18,575** | |
|  | *Mean (SD)* | 30.42 | 6.9 | 27.35 | 5.4 | 27.70 | 5.8 |
| Situation level characteristics | | | | | | | |
| Area |  | **n = 2,806** | | **n = 20,978** | | **n = 23,784** | |
|  | *City* | 405 | 14% | 2,769 | 13% | 3,174 | 13% |
|  | *Town or suburb* | 1,303 | 46% | 9,569 | 46% | 10,872 | 46% |
|  | *Rural* | 1,098 | 39% | 8,640 | 41% | 9,738 | 41% |
| Household income |  | **n = 1,793** | | **n = 15,148** | | **n = 16,941** | |
|  | *Up to £22,425* | 767 | 43% | 5,054 | 33% | 5,821 | 34% |
|  | *£22,425 - £37,375* | 599 | 33% | 4,758 | 31% | 5,357 | 32% |
|  | *£37,375 +* | 427 | 24% | 5,336 | 35% | 5,763 | 34% |
| WIMD |  |  |  | **n = 22,885** | | **n = 25,827** | |
|  | *80-100% most deprived* | 472 | 16% | 2,663 | 12% | 3,135 | 12% |
|  | *60-80%* | 700 | 24% | 6,282 | 27% | 6,982 | 27% |
|  | *40-60%* | 648 | 22% | 4,876 | 21% | 5,524 | 21% |
|  | *20-40%* | 576 | 20% | 3,615 | 16% | 4,191 | 16% |
|  | *0-20% least deprived* | 546 | 19% | 5,449 | 24% | 5,995 | 23% |
|  |  |  |  |  |  |  |  |

Appendix 7: Multilevel model comparisons for wellbeing outcome models

To assess the extent of clustering we compared the fit of three multilevel regression models: clustering at GP practice level only (M1); clustering at Health Board level only (M2); GP clustered within Health Board (M3) to a standard regression model (M0). These models include the variable of interest (confidence to self-manage) as the only fixed-effect (see Table below for model fit statistics).

**Model fit statistics for the fixed effects and multilevel models (N = 2615)**

| Statistic | Fixed effects only  M0 | Mixed effects M1 (GP only) | Mixed effects M2 (health board only) | Mixed effects M3 (GP & health board) |
| --- | --- | --- | --- | --- |
| AIC | 23695.99 | 23689.65 | 23692.08 | 23689.22 |
| BIC | 23713.60 | 23713.12 | 23715.56 | 23718.57 |
| R^2^ | 0.17 | 0.19 | 0.17 | 0.19 |
| ICC | - | 0.02 | 0.004 | 0.02 |

Note. AIC = Akaike Information Criterion, BIC = Bayesian Information Criterion, ICC = Intra-class correlation

As seen in the table above, model fit statistics indicated that including GP practice level improved model fit, but that including health board did not provide additional information. A likelihood ratio (LR) test (between M1 and M3) indicated that a multilevel structure with GP/Health Board did not improve the model fit (χ² (1) = 1.79, *p* = 0.18). As a result, the final model in this study, was a multilevel regression model with nesting at GP level only. See appendix 7 for the final model results.

Appendix 8: Wellbeing: multilevel Linear regression model results

|  |  | Model results | | |
| --- | --- | --- | --- | --- |
| *Predictors* |  | ***B*** | ***CI*** | ***p*** |
| (Intercept) |  | 32.10 | 27.74 – 36.47 | **<0.001** |
| Confidence to self-manage | *Per increase of 1* | 12.36 | 11.13 – 13.60 | **<0.001** |
| Gender | *Female* | *Ref.* | | |
|  | *Male* | 4.77 | 3.17 – 6.37 | **<0.001** |
| Age | *Per 1 category increase* | 0.05 | 0.01 – 0.09 | **0.02** |
| Education | *No formal qualifications* | *Ref.* | | |
|  | *GCSE/NVQ/equivalent* | 0.87 | -1.29 – 3.02 | 0.43 |
|  | *A-level, degree or higher/equivalent* | 1.78 | -0.23 – 3.79 | 0.08 |
| Employment status | *Employed* | *Ref.* | | |
|  | *Retired* | 1.91 | -0.44 – 4.26 | 0.11 |
|  | *Unable to work due to sickness or ill-health* | -15.74 | -18.86 – -12.62 | **<0.001** |
|  | *Other* | -5.23 | -9.67 – -0.79 | **0.02** |
| Chronic conditions | *1* | *Ref.* | | |
|  | *2* | -3.28 | -5.96 – -0.59 | **0.02** |
|  | *3+* | -15.77 | -18.29 – -13.26 | **<0.001** |
| LSOA WIMD | *80-100% most deprived* | *Ref.* | | |
|  | *60-80%* | 3.68 | 1.03 – 6.32 | **<0.01** |
|  | *40-60%* | 0.87 | -1.77 – 3.51 | 0.52 |
|  | *20-40%* | 0.15 | -2.51 – 2.80 | 0.91 |
|  | *0-20% least deprived* | 3.31 | 0.51 – 6.11 | **0.02** |
| Random Effects |  | | | |
| σ^2^ |  | 399.48 | | |
| τ00 |  | 5.45 | | |
| ICC |  | 0.01 | | |
| N |  | 199 | | |
| Observations |  | 2600 | | |
| Marginal R^2^/Conditional R^2^ |  | 0.335   / 0.344 | | |

*Note*. *B* = regression coefficient, CI = 95% confidence interval, *p* = p-value (bold highlight indicates less than .05 significance level). σ2= variance of residuals, τ00 = variance of random intercept, ICC = Intra-class Correlation.

Appendix 9: Emergency admissions: Logistic regression model results

|  |  | Model results | | |
| --- | --- | --- | --- | --- |
| *Predictors* |  | **OR** | ***CI*** |  |
| (Intercept) |  | 0.03 | 0.02 - 0.07 |  |
| Confidence to self-manage | *Per increase of 1* | **0.80** | **0.66 - 0.96** |  |
| Gender | *Female* | *Ref.* | | |
|  | *Male* | 1.32 | 1.03 - 1.70 |  |
| Age | *Per 1 category increase* | **1.02** | **1.01 - 1.02** |  |
| Education | *No formal qualifications* | *Ref* | | |
|  | *GCSE/NVQ/equivalent* | 1.16 | 0.83 - 1.61 |  |
|  | *A-level, degree or higher/equivalent* | 1.34 | 1.00 - 1.82 |  |
| Employment status | *Employed* | *Ref.* | | |
|  | *Retired* | 1.02 | 0.69 - 1.53 |  |
|  | *Unable to work due to sickness or ill-health* | **2.52** | **1.60 - 3.98** |  |
|  | *Other* | 0.63 | 0.21 - 1.50 |  |
| Chronic conditions | *1* | *Ref.* | | |
|  | *2* | 1.54 | 0.93 - 2.65 |  |
|  | *3+* | **1.97** | **1.24 - 3.29** |  |
| LSOA WIMD | *80-100% most deprived* | *Ref.* | | |
|  | *60-80%* | 0.93 | 0.62 - 1.40 |  |
|  | *40-60%* | 1.12 | 0.75 - 1.68 |  |
|  | *20-40%* | 1.30 | 0.88 - 1.95 |  |
|  | *0-20% least deprived* | 1.07 | 0.70 - 1.65 |  |
| Observations  AIC |  | 2600  1817.65 | | |
| PCP |  | 0.80 | | |

*Note*. OR = odds ratio, CI = 95% confidence interval, (bold highlight indicates CI does not cross 1), AIC = Akaike Information Criterion, PCP = Percentage of Correct Predictions.

Appendix 10: Elective admissions: Logistic regression model results

|  |  | Model results | | |
| --- | --- | --- | --- | --- |
| *Predictors* |  | **OR** | ***CI*** |  |
| (Intercept) |  | 0.07 | 0.04 -0.13 |  |
| Confidence to self-manage | *Per increase of 1* | 1.04 | 0.89 -1.23 |  |
| Gender | *Female* | *Ref.* | | |
|  | *Male* | 1.23 | 0.99 -1.53 |  |
| Age | *Per 1 category increase* | **1.00** | **1.00 -1.01** |  |
| Education | *No formal qualifications* | *Ref* | | |
|  | *GCSE/NVQ/equivalent* | 0.90 | 0.68 -1.21 |  |
|  | *A-level, degree or higher/equivalent* | 1.04 | 0.80 -1.36 |  |
| Employment status | *Employed* | *Ref.* | | |
|  | *Retired* | 0.93 | 0.68 -1.28 |  |
|  | *Unable to work due to sickness or ill-health* | **1.49** | **1.01 - 2.19** |  |
|  | *Other* | 0.68 | 0.32 -1.30 |  |
| Chronic conditions | *1* | *Ref.* | | |
|  | *2* | 1.48 | 0.96 -2.34 |  |
|  | *3+* | **2.51** | **1.69 -3.86** |  |
| LSOA WIMD | *80-100% most deprived* | *Ref.* | | |
|  | *60-80%* | 1.14 | 0.81 -1.62 |  |
|  | *40-60%* | 1.08 | 0.77 -1.54 |  |
|  | *20-40%* | 1.07 | 0.75 -1.52 |  |
|  | *0-20% least deprived* | 1.21 | 0.84 -1.75 |  |
| Observations  AIC |  | 2600  2298.16 | | |
| PCP |  | 0.73 | | |

*Note*. OR = odds ratio, CI = 95% confidence interval, (bold highlight indicates CI does not cross 1), AIC = Akaike Information Criterion, PCP = Percentage of Correct Predictions.
